# Evaluation of a novel home-based laparoscopic and core surgical skills programme (Monash Online Surgical Training)

**DOI:** 10.1007/s00464-023-10669-8

**Published:** 2024-02-01

**Authors:** Samantha Leng, Noor Chaudhry, Maurizio Pacilli, Ramesh Mark Nataraja

**Affiliations:** 1 Department of Paediatric Surgery & Monash Children’s Simulation, Monash Children’s Hospital, Melbourne, Australia; 2 Department of Paediatrics, Faculty of Medicine, Nursing and Health Sciences, School of Clinical Sciences, Monash University, Melbourne, Australia; 3 Department of Surgery, Faculty of Medicine, Nursing and Health Sciences, School of Clinical Science, Monash University, Melbourne, Australia

**Keywords:** Laparoscopic simulation, Simulation-based education, Surgical training, Laparoscopic bench trainers, Home-based education

## Abstract

**Introduction:**

Limitations to surgical education access were exacerbated during the COVID-19 Pandemic. In response, we created a national home-based comprehensive surgical skills course: Monash Online Surgical Training (MOST). Our aim was to evaluate the educational impact of this approach.

**Methods:**

A remote, 6-week course was designed with learning objectives aligned to the national surgical training. Participants received a personal laparoscopic bench trainer, instrument tracking software, live webinars, access to an online theoretical learning platform, and individualised feedback by system-generated or expert surgeons’ assessments. Mixed method analysis of instrument tracking metrics, pre- and post-course questionnaires (11 core surgical domains) and participant comments was utilised. Data were analysed using the Mann–Whitney *U* test, and a *p*-value of < 0.05 was considered statistically significant.

**Results:**

A total of 54 participants with varied levels of experience (1 to > 6 years post-graduate level) completed MOST. All 11 learning-outcome domains demonstrated statistically significant improvement including core laparoscopic skills (1.4/5 vs 2.8/5, *p* < 0.0001) and handling laparoscopic instruments (1.5/5 vs 2.8/5, *p* < 0.0001). A total of 3460 tasks were completed reflecting 158.2 h (9492 min) of practice, 394 were submitted for formal feedback. Participants rated the course (mean 8.5/10, SD 1.6), live webinars (mean 8.9/10, SD 1.6) and instrument tracking software (mean 8.6, SD 1.7) highly. Qualitative analysis revealed a paradigm shift including the benefits of a safe learning environment and self-paced, self-directed learning.

**Conclusion:**

The MOST course demonstrates the successful implementation of a fully remote laparoscopic simulation course which participants found to be an effective tool to acquire core surgical skills.

**Graphical abstract:**

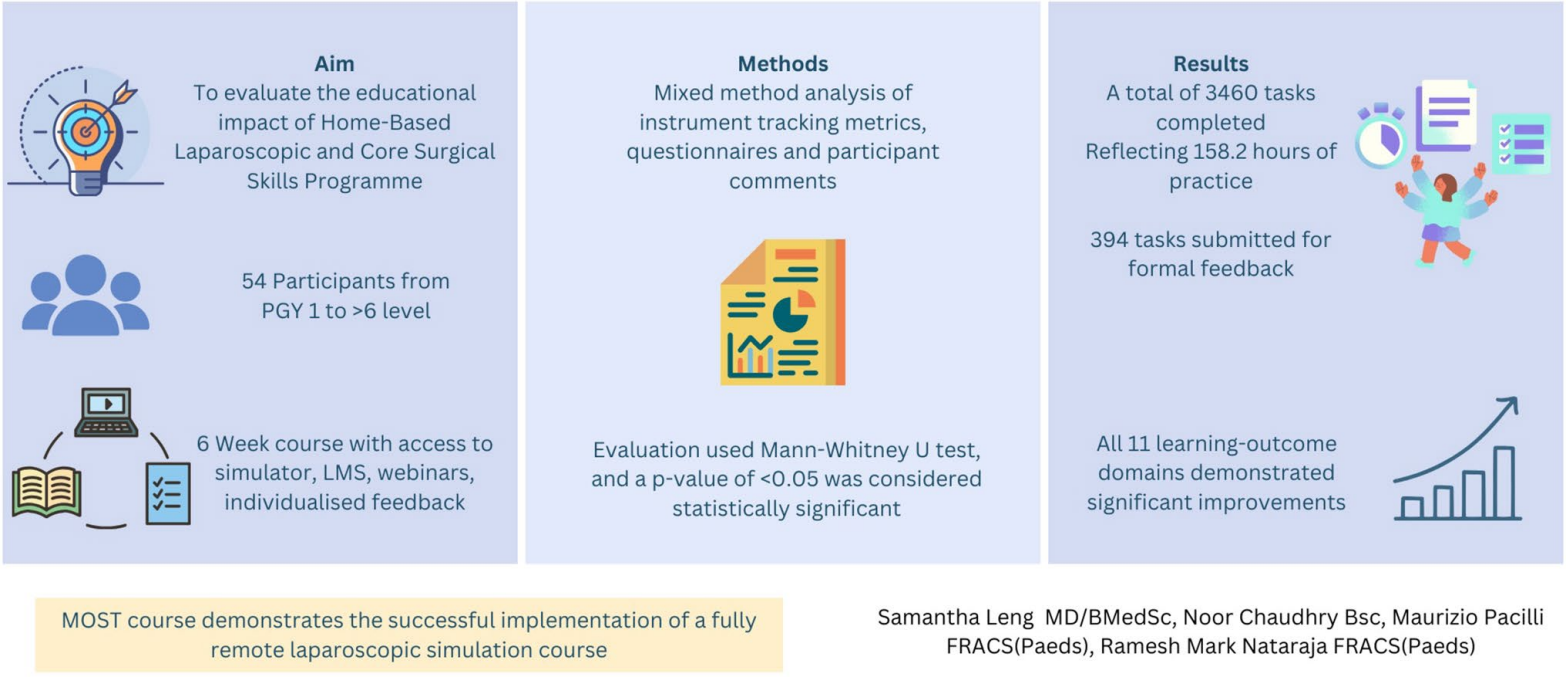

Historically, surgical training has followed an apprenticeship model, where technical skills are acquired as a direct result of clinical exposure under the supervision of a trained surgeon [1, 2]. This traditional Halstedian model of time-bound apprenticeship has now been replaced with competency-based training in many surgical training programmes [1, 3]. With the increased utilisation of laparoscopic surgery, surgical trainees are now required to master an entirely different skill set in addition to that of open surgery [4]. Laparoscopic surgery requires altered hand-eye co-ordination, 2D-to-3D perception realization, increased fine motor skills, adaptation to the fulcrum effect of the patient’s body wall and the effects of the loss of haptic feedback [5]. These skills can all be acquired in a simulation-based educational environment prior to patient contact which, apart from the inherent patient safety aspects, also has various learner centric advantages.

Systematic reviews have previously demonstrated the benefits of simulation-based education, both via virtual reality and box training modalities, in the acquisition of laparoscopic skills [6]. However, limited access to costly equipment and time constraints often act as barriers [7]. This is particularly true when simulation-based education is confined to dedicated facilities, creating a paucity of access for participants in both high and low-middle income (LMIC) settings [8, 9]. There are additional challenges of simulation-based education opportunities in certain specialities or task trainers designed to accurately simulate the size constraints and rare clinical conditions [10].

The COVID-19 pandemic posed additional challenges for surgical trainees on an international scale with national lockdowns and travel restrictions. This limited exposure to laparoscopic training, both due to a reduction in elective operating and by creating a further barrier to in-person education or academic opportunities [11]. In response, we devised a programme with a low-cost simulator, that allowed participants access to self-directed core surgical and laparoscopic skills acquisition in their own home environment; the Monash Online Surgical Training (MOST) course.

Our aims were to evaluate the implementation of this fully remote laparoscopic simulation course, and its educational impact by participants in the development of core laparoscopic skills.

## Materials and methods

### Course structure

The MOST course is a 6-week, fully remote, home-based laparoscopic simulation course, accredited for 6 credit points towards the Graduate Certificate of Clinical Simulation at Monash University or undertaken by participants as a stand-alone programme. The curriculum was adapted from an existing core surgical skills course that had been delivered for many years in a traditional face-to-face format. The courses’ intended learning objectives (ILOs) were developed from the requirements for core surgical training determined by the Royal Australasian College of Surgeons [12]. Targeted learners were junior doctors with an interest in surgical specialties that utilise laparoscopy such as general surgery, paediatric surgery, gynaecology and urology, who had completed at least 3 to 4 years of post-graduate training. Participants enrolled in the course voluntarily as an additional opportunity for learning outside of their clinical practice and training requirements.

The Moodle learning platform (Moodle version 3.9.17+, Perth Western Australia) was used to deliver theoretical content, which participants worked through at their own pace. The curriculum revolved around the essentials of providing surgical care and included learning topics titled “the fundamentals of surgery”, “prepping and draping for surgical procedures”, “consent issues”, “ethical dilemmas”, “handling of laparoscopic and open instruments”, “optimisation of risk factors” and “stabilisation of patients pre-operatively”. Other domains included communication and cognitive reasoning skills, academic principles such as “manuscript writing”, “crafting Curriculum Vitae” and “preparing for interviews”. Discussion forums were an additional feature of this learning platform and facilitated conversations between participants in the course and expert surgeons. The forums were moderated by faculty and tested the higher order thinking of the participants.

Multimodal educational content was delivered over weekly live webinars through the Zoom video conferencing platform (Zoom version 5.13.11, Zoom Video Communications, San Jose California USA) by one to two experienced surgeons acting as facilitators. Each virtual tutorial was approximately 90 min in duration. The surgeons, who were consultants in paediatric surgery, provided expert opinions during discussion sessions and facilitated live laparoscopic training. Participants were able to join the sessions nationally from a venue of their choice (such as from home or their workplace). During these webinars, participants shared the inner view of their laparoscopic simulator so that their performance could be viewed by the other participants and the faculty. The ratio of faculty member to participants in these webinars was intentionally high to maintain optimal learner engagement in the educational session.

### Laparoscopic simulator

**Fig. 1:**
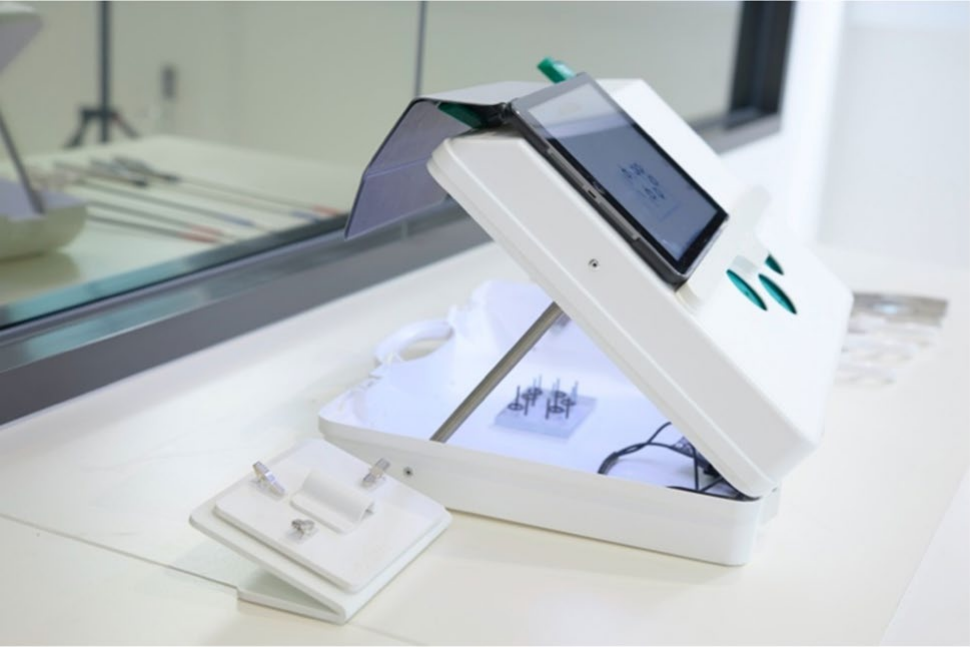
The eoSim SurgTrac Core laparoscopic simulation bench trainer (eoSurgical Ltd, Scotland, UK) used with an iPad Tablet (Apple Inc, Cupertino, California, USA)

The eoSim SurgTrac Core surgical simulator (eoSurgical Ltd., Scotland UK) and associated SurgTrac instrument tracking cloud-based software (SurgTrac version 1.9.9 Windows and MacOS, eoSurgical, Scotland UK) were selected for the MOST course. The eoSim device was determined to be cost-effective, portable and had been previously validated for simulation-based laparoscopic skills training [13, 14] (Fig. 1). Participants received all the laparoscopic instruments (ratcheted grasper, non-ratcheted grasper, scissors), as well as the task materials required to complete the laparoscopic exercises (such as peg board, thread, precision cutting board). Participants practised seven core tasks to emulate technical skills required for laparoscopic surgery including “thread transfer”, “precision cutting”, “dice stacking”, “paper folding”, “peg capping”, “untangling a paper clip” and “intracorporeal suture and tie”. Participants were able to complete simulation tasks in their own time, and there was no limit to the number of practice trials. Participants also had the option of submitting videos of their simulation attempts for review by expert surgeons in order to receive personalised feedback. Performance metrics were automatically generated and recorded by the SurgTrac instrument tracking software. These metrics included “overall time taken”, “time spent off-screen”, “speed and acceleration”, “distance travelled”, “handedness and motion smoothness” of the laparoscopic instruments. The above performance metrics were available for participants to review on their devices on completion of the exercise to guide ongoing self-directed practice. The SurgTrac software was additionally utilised to capture data including the number of tasks performed during each course period and the number of tasks submitted for final assessment.

### Laparoscopic assessment

**Fig. 2:**
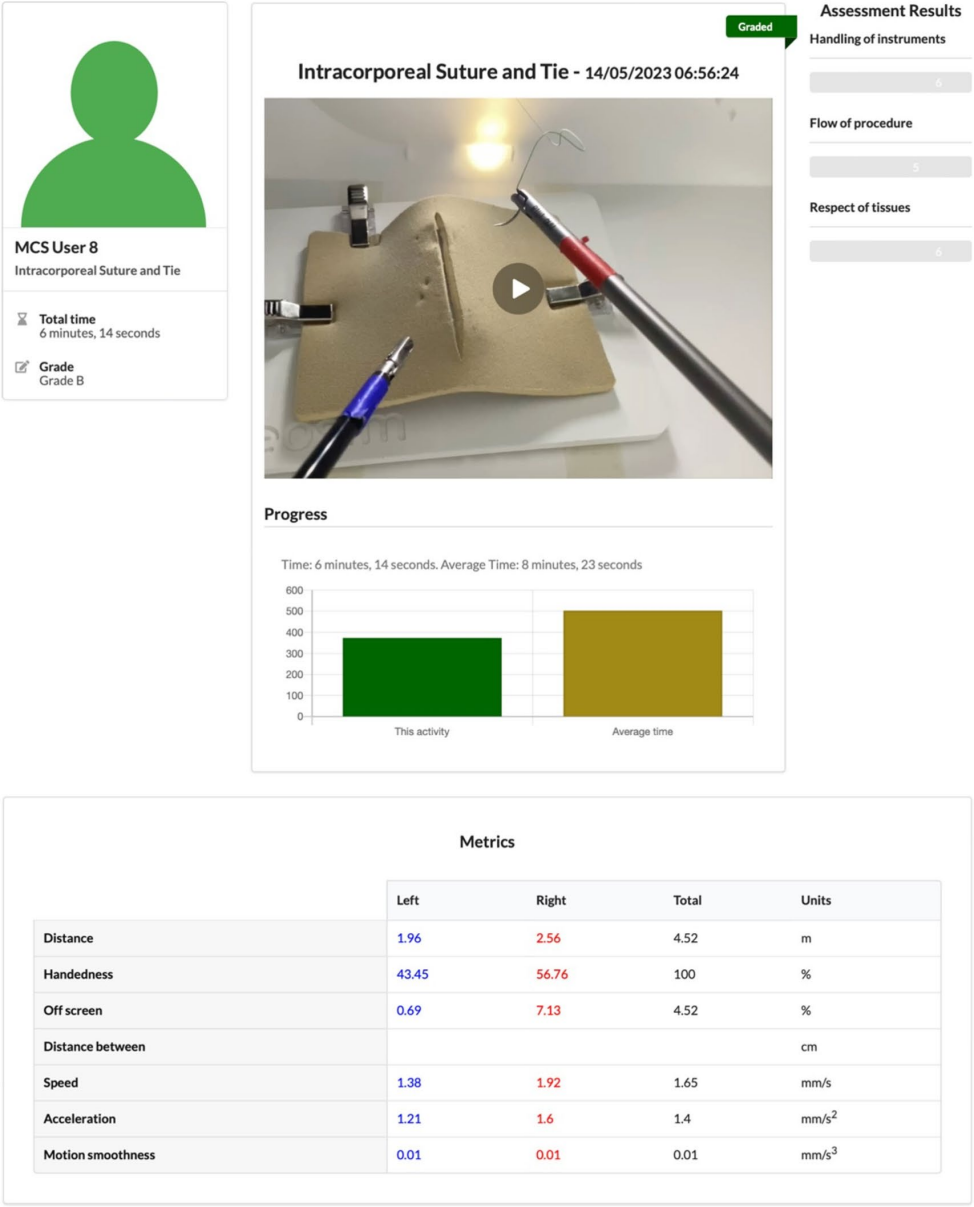
Example of the feedback template for the assessment task and metrics on SurgTrac, version 1.9.9, eoSurgical, Scotland UK

Participants were asked to submit video recorded attempts at laparoscopic exercises to faculty members for review and formal assessment. A minimum number of six core and one intra-corporeal suturing assessments were required to be submitted for successful course completion. However, participants were encouraged to submit additional attempts. Performance metrics that had been generated by the SurgTrac software, as well as the video of the task itself, were reviewed by faculty. Their assessment was presented to the participant in the form of an overall grade as well as constructive comments. Figure 2 demonstrates an example of a participants graded laparoscopic skills task.

### Course fees and cost

The course fees were determined according to Monash University guidelines and included faculty, equipment, postage costs and course materials. The standard fee was $4,500 Australian dollars (AUD). Discounts were available for participants linked to certain institutions. The participants received lifetime access to the SurgTrac software licence and retained the eoSim box trainer on completion of the course, which would otherwise individually retail for $2,000 AUD each. For a cohort of ten participants, the average total cost of running the MOST course over 6 weeks was $29,131 AUD which was inclusive of faculty time (administrative and academic), purchase of the simulators and required equipment, packaging with shipping fees, and learning management systems access. Most of these costs were attributed to purchase of the discounted eoSim box trainers ($15,000 AUD) and international shipping ($1250 AUD). Faculty members spent an estimated total of 93 h with course planning and preparation, assessment and facilitating webinars for each intake. The total cost attributed to faculty time was approximately $11, 481 AUD and administration costs $1400 AUD.

### Data collection & analysis

Both qualitative and quantitative voluntary feedback were obtained from the participants who were aware of the requirement to evaluate this novel programme. Qualitative feedback was sought in the form of comments provided within pre-course and post-course questionnaires in 11 domains. Thematic analysis was used to analyse the transcripts as per the work of Braun and Clarke [15]. Quantitative feedback was also obtained from questionnaires in the form of Likert scale data. Qualtrics (Qualtrics XM, version May 2023, Provo Utah USA) was utilised for anonymous pre-course and post-course evaluation surveys. Demographic data, including trainee age, gender, occupation, post-graduate level, previous experience performing, assisting and observing laparoscopic surgery, were collected during the pre-course survey. Participants were also asked to rate their confidence level prior to commencing the MOST course on a five-point Likert scale for each ILO domain. These included

1. Knowledge of laparoscopic principles.
2. Core laparoscopic skills.
3. Intra-corporeal knot tying.
4. Arranging urgent theatre cases.
5. Preparing, draping, and operating room ergonomics.
6. Identifying and handling open surgical instruments.
7. Handling of common laparoscopic instruments.
8. Writing operative notes.
9. Conducting daily ward rounds of surgical patients.
10. Obtaining informed consent for minor procedures.
11. Dealing with challenging situations on the ward.

In the post-course evaluation survey, participants were asked to complete the same confidence scales as well as provide feedback on each educational modality of the MOST course, inclusive of discussion webinars, live laparoscopic webinars, online learning resources, use of the SurgTrac software and bench trainer, online task feedback from SurgTrac assessments. An 11-point Likert scale was utilised, in which zero signified ‘not useful’ and ten signified ‘most useful’ for each domain. Participants were also asked to provide an overall course rating.

### Statistical analysis

Statistical analysis was performed using GraphPad Prism (GraphPad Prism version 9.0, GraphPad Software, La Jolla California USA). The Kolmogorov–Smirnov test was used to determine the normality between relevant pre- and post-confidence ratings. The Mann–Whitney *U* test was used to determine the difference between pre- and post-confidence levels in the 11 domains as distribution was ordinal but not normally distributed. *P*-values of less than or equal to 0.05 were considered statistically significant.

## Results

### Participant demographics

Between 2020 and 2022 five cohorts of participants completed the MOST course. A total of 54 participants completed the course out of which 46 responded to the pre-course evaluation survey and 32 responded to the post-course evaluation survey. Some participants chose to omit responses to several survey questions: pre-course 10.9% (5/46), post-course 3% (1/32). An average of 11 participants attended each course. Fifteen (33.3%) participants had applied, or were interested in applying, for the national accredited surgical training programme in the year they completed the course. Interest in specialties varied across the cohorts with most participants interested in General Surgery, 64.3% (27/42). Participants had different amounts of prior exposure to laparoscopic surgery before commencing the MOST course. All 42 participants had observed a laparoscopic procedure, 90.5% had assisted in one or more laparoscopic procedures and 61.9% of participants had performed one or more laparoscopic procedures as the primary operator, Table 1.

**Table 1:** Trainee demographics (obtained from pre-course evaluation survey)

Participant demographics	% (*n*)
Gender	
Male	37% (17/46)
Female	63% (29/46)
Age	
20–24 years	2.2% (1/45)
25–29 years	55.5% (25/45)
30–34 years	31.1% (14/45)
> 35 years	11.1% (5/45)
Post graduate year	
PGY 1	2.2% (1/45)
PGY 2	22.2% (10/45)
PGY 3	22.2% (10/45)
PGY 4	11.1% (5/45)
PGY 5	22.2% (10/45)
PGY 6+	20.0% (9/45)
Specialty interest	
General surgery	64.3% (27/42)
Paediatric surgery	11.9% (5/42)
Gynaecology	16.6% (7/42)
Vascular	2.4% (1/42)
Urology	2.4% (1/42)
ENT	2.4% (1/42)
Applying for SET* training	
Yes	33.3% (15/45)
No	66.6% (30/45)
Prior experience in laparoscopic surgery	
Observed	100% (42/42)
Assisted	90.5% (38/42)
Performed	61.9% (26/42)

*The Surgical Education and Training (SET) refers to the national accredited surgical training programme

### Course evaluation

**Fig. 3:**
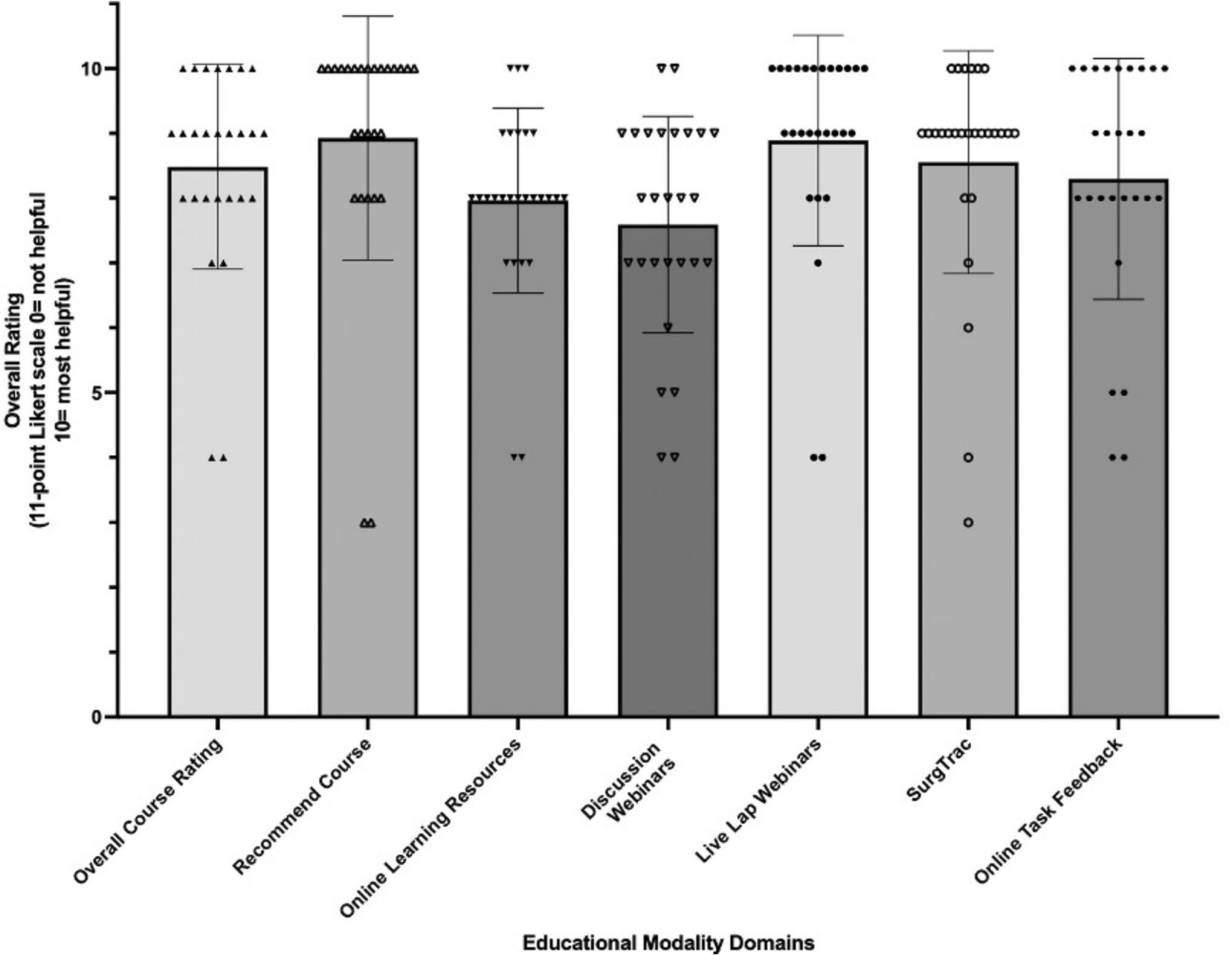
Trainee ratings of individual aspects of the MOST Course (data obtained from post-course evaluation surveys)

In all eleven ILO domains, participants demonstrated a statistically significant improvement across the five courses, Table 2*.* Overall, participants rated the course highly, giving a mean rating of 8.5 (SD 1.6) out of 10 across all domains. Live laparoscopic webinars and the integrated SurgTrac software were rated most highly at 8.9 (SD 1.6) and 8.6 (SD 1.7), respectively. Participants benefited least from the discussion webinars which were rated a mean of 7.6 (1.6) (Fig. 3).

**Table 2:** Comparison of the participants’ pre-course vs. post-course confidence rating for each educational learning-outcome domain

Educational domain	Domain rating by participants mean (SD), 0–5 Likert Scale
Knowledge of laparoscopic principles	1.7 (0.9) vs 2.7 (0.8), *p* < 0.0001*
Core laparoscopic skills	1.4 (0.8) vs 2.8 (0.8), *p* < 0.0001*
Intra-corporeal knot tying	0.6 (0.7) vs 2.2 (0.9), *p* < 0.0001*
Arranging urgent theatre cases	2.7 (1.0) vs 3.3 (0.6), *p* = 0.0004*
Preparing, draping, and operating room ergonomics	2.2 (1.0) vs 3.2 (0.5), *p* < 0.0001*
Identifying and handling open surgical instruments	2.2 (1.2) vs 3.0 (0.6), *p* < 0.0001*
Handling of common laparoscopic instruments	1.5 (0.9) vs 2.8 (0.7), *p* < 0.0001*
Writing operative notes	2.1 (1.0) vs 3.3 (0.6), *p* < 0.0001*
Conducting daily ward round of surgical patients	2.9 (0.7) vs 3.6 (0.6), *p* < 0.0001*
Obtaining informed consent for minor procedures	3.0 (0.8) vs 3.6 (0.5), *p* < 0.0003*
Dealing with challenging situations on the ward	2.7 (0.7) vs 3.2 (0.6), *p* < 0.0001*

### Laparoscopic task completion

Across the five courses, 3460 tasks were completed by participants, spanning a duration of 158.2 h or 9492 min. Each trainee performed an average of 64 laparoscopic skills tasks across the 6-week course period, constituting 2.9 h of practice. A total of 394 tasks (20.6 h duration) were video recorded and submitted for review and assessment by an expert surgeon. This only included activity that was performed using the SurgTrac software.

### Participant feedback

Two predominant positive themes were noted during thematic analysis of free text written feedback from *simulation-based education* participants. Firstly, participants identified that the MOST course supported them as an individual learner. This was applicable to both self-directed learning opportunities, as well as targeted and individualized feedback on task performance. Secondly, the importance of a safe learning environment, in the absence of external judgement or a time-pressure, was noted by participants to be a benefit of the MOST course. Regarding areas for improvement, the broad nature of the theoretical components of the course design and repetition of academic material also seen in similar alternative courses was mentioned most frequently, Table 3.

**Table 3:** Major themes obtained from the thematic analysis of participants written feedback, with illustrative direct participant comment

Major themes	Participant comments
Focusing on the participant’s individual learning needs	Participant 1, November 2020: “As I’ve been on nights the whole time this course has been running, theatre access has been very hit and miss. This course has been my theatre practice whenever I’ve had time. It’s been a good substitute for being in the operating theatre. I haven’t had the opportunity to practice these skills in theatre. It is a great tool to keep your skills going, and I haven’t felt like I’ve lost any skills. This course is a good substitute to keep my skills current” Participant 11, November 2020: “The individualised feedback (was the most useful) …” Participant 1, November 2020: “In regard to research and career planning—I found the content really really good. I’ve heard people talk about how to build up your CV, but it was really good to go through it point by point. All the interview prep was broken down into questions; how to target and practice specific things was really useful”
Benefits of an active learner with a practical skills focussed learning experience with provision real time constructive feedback	Participant 4, November 2020: “The hands-on practice with the lap trainer with feedback from an experienced operator in real time” Participant 10, November 2020: “Interactive sessions and live feedback on laparoscopic skills” Participant 1, March 2021: “Feedback from SurgTrac, and especially live webinars offering contemporaneous feedback and tips” Participant 9, November 2021: “… getting feedback and tips from an experienced operator in real time” Participant 1, March 2022: The live skills sessions were crucial for tips on how to be successful with practice and the SurgTrac application and being able to see your progress is very enjoyable” Participant 2, October 2022: “…. online laparoscopic webinars (were the most useful) with live demonstration, and feedback”
Positive aspects of creating a safe learning environment	Participant 2, November 2020: “Thorough explanation of the principles and basics of laparoscopic surgery in a friendly environment with no time pressure”
	Participant 5, March 2021: “…practicing with the instruments in a safe and non-judgemental zone (i.e., at home) so that I can finally start doing more in theatre and be taken more seriously by my seniors”
Assigning value to a home simulation-based educational environment	Participant 4, October 2022: “Able to practise lap surgery in my own time without any pressure” Participant 2, November 2020: “Focussing on repetition of the fundamental tasks was really helpful to getting core laparoscopic skills right” Participant 2, October 2022: “Easily accessible from home (was the most useful part)”
Repetition of core areas and broad or repetitive learning modalities	Participant 5, September 2021: “Course chapters that could be found online and in textbooks elsewhere (were the least useful)” Participant 4, November 2020: “The essay assessment (was the least useful)—I've had to do SO much of this (no fault of the course, it's just very en vogue at Uni, RACS courses and my Masters)” Participant 2, October 2022: “participation with online discussion forums may be limited by the fact that course material was completed at different paces...might be better to allocate small section of online webinar to have a live discussion”

## Discussion

The MOST course has demonstrated that a home-based core surgical and laparoscopic skills training course is not only feasible but also effective, for novice participants to acquire laparoscopic operating skills. Previous studies have demonstrated that simulation training is an effective method by which participants can acquire experience in laparoscopic and other minimally invasive surgical techniques, but these were not in a home-based environment [16, 17].

Participants reported a statistically significant increase in their confidence for all 11 of our learning domains on completion of the 6-week programme, which included both hands-on skills with laparoscopic instruments as well as essential knowledge for a surgical trainee in daily clinical practice. These were confirmed in the thematic analysis as examples of the positive learning experiences that were experienced by the participants.

In the Australian training context, there is no formal requirement for trainees to participate in simulation prior to, or in conjunction with, clinical exposure to laparoscopic operating at the pre-vocational level. This differs from the Scandinavian or American training environments, for example, in which many trainees must undertake mandatory simulation practice [18, 19]. Further, during the COVID pandemic, trainee exposure to laparoscopic surgery in the clinical setting was affected by the reduction in the number of elective operations and cancellation of in-person laparoscopic skills workshops [11]. As a result, barriers to skill acquisition such as trainee time constraints, access to equipment and difficulty obtaining directive feedback were exacerbated [20]. These barriers were potentially compensated by the MOST course as two repeatedly identified benefits included increased access to hands-on laparoscopic skills practice and individualized feedback from experts. The fully remote course structure allowed participants to learn basic laparoscopic skills at their own convenience. Participants were able to perform as many task repetitions as they required and progressed through more difficult tasks at their own pace. This incorporates the learning theories of Deliberate Practice and Mastery Learning and is in keeping with a review by Ericsson et al that determined having a specific defined task, detailed immediate feedback and opportunity for repetition were all factors required for satisfactory acquisition of a new skill [21, 22].

Access to laparoscopic bench trainers at home was a particular benefit raised by participants who had limited exposure during their clinical practice. For example, one participant commented that they had minimal operating theatre access as they were working an extended period of night shifts. It is possible to extrapolate that this positive aspect of the MOST course, as a novel form of home and simulation-based education, would also translate to uncommonly performed operations for the expert surgeon.

Thematically, the benefits of the MOST course identified by participants can be summarised into consideration of the individual’s learning needs, and the value of a safe learning environment. Participants recognised that the home-based design and feedback methodology allowed the MOST course to be tailored to the needs of each individual learner. With the limitations of scheduling and access to simulation equipment removed, participants commented that they were able to “focus on repetition of fundamental tasks” at their own pace. Additionally, the merits of self-directed learning were further bolstered by the individualised feedback component of MOST. This is consistent with the results found in a previous qualitative review of home-based simulation training by Blackhall et al. which identified the absence of direct feedback as a significant barrier for participants [20]. Automated metric feedback from SurgTrac, and our faculty members in the form of task grading as well as during live webinars were highlighted by 12 participants as one of the most valuable components of the course. The analysis of these system-generated metric is beyond this scope of this current study. Further, several participants identified that the home-based simulation environment allowed them to focus on technical practice in a low-pressure environment.

Trainee participation in pre-course and post-course evaluation surveys posed a limitation on our ability to accurately evaluate the perceived usefulness of our laparoscopic skills course. Although 85% of participants completed the pre-course survey, only 55.6% completed the post-course survey, providing data on their confidence with laparoscopic skills and their overall rating of the course structure and content. Given our small cohort of 54 total participants, this could potentially introduce bias. We recognise that inclusion of additional methods of data collection, with either interview-based discussions or clinical outcome capture (such as in the form of operating logbooks), would be useful to further verify the participants’ reported views. However, as the participants were from different states, working in different health networks and at different post-graduate levels, there are logistic and data interpretation challenges with capturing this clinical data.

Previous United Kingdom and Ireland-based studies have demonstrated that uptake of home-based laparoscopic skills training programs is generally poor compared to that of in-person short courses [20]. This may be a product of perceived effort and commitment, of both time and finances, required to complete a more comprehensive home-based training programme. Our results are not dissimilar, demonstrating that even over the course of 6 weeks, participants only formally practised an average of 2.9 h each on the SurgTrac system. This raises the concerns regarding whether the MOST course, or a similar remote-based course design, is the most cost-effective strategy for simulation-based education. We were not able to assess any time participants may have spent utilising the box trainers without use of the SurgTrac software, nor are we able to capture any further practice participants have undertaken on completion of the MOST course. However, a Scandinavian study has previously identified that access to box trainers is not the limiting factor that prevents many doctors from practicing their laparoscopic skills. [18] Thus, it should be considered whether a mandatory home simulation-based programme may be more effective.

Negative themes identified in our cohort of MOST course participants might indicate that junior surgical trainees are exposed to a multitude of academic responsibilities outside of the clinical setting. Further, educational content within the MOST course that was deemed to be a repetition of other coursework, or too broad in nature was looked unfavourably upon by participants.

As a relatively low-cost, fully remote programme, the MOST course has potential value as a tool in other settings where access to laparoscopy in a clinical setting or simulation lab is limited. It is still an ongoing course with current enrolments at our institution. Similar remote surgical skills programs have previously been implemented in countries such as Bolivia, Mexico and Argentina, and have demonstrated positive results with surgeons reporting increased confidence in performing procedures such as laparoscopic cholecystectomies [8]. As such, this positive result might also be demonstrated in rural or LMIC regions in the Asia-Pacific region.

## Conclusions

The MOST course has demonstrated that it is feasible to develop a fully remote and home-based laparoscopic skills training programme with bench trainers. Participants perceived the 6-week course to be helpful in all evaluated domains, including hands-on skills and academic training modules. Following the COVID era, the MOST course continues to run at our institution in a fully remote format.

Future iterations of the MOST course may also be applicable to rural or low-middle-income settings as an alternative to simulation lab training. Further evaluation into the validity of the quantitative SurgTrac data, and its translation to participants’ competency in clinical practice would be valuable.
